# 5-(4,4′′-Difluoro-5′-hy­droxy-1,1′:3′,1′′-terphenyl-4′-yl)-3-(morpholin-4-ylmeth­yl)-1,3,4-oxadiazole-2(3*H*)-thione

**DOI:** 10.1107/S1600536811048471

**Published:** 2011-11-19

**Authors:** Hoong-Kun Fun, Suhana Arshad, S. Samshuddin, B. Narayana, B. K. Sarojini

**Affiliations:** a X-ray Crystallography Unit, School of Physics, Universiti Sains Malaysia, 11800 USM, Penang, Malaysia; b Department of Studies in Chemistry, Mangalore University, Mangalagangotri 574 199, India; c Department of Chemistry, P. A. College of Engineering, Nadupadavu, Mangalore 574 153, India

## Abstract

In the title compound, C_25_H_21_F_2_N_3_O_3_S, the morpholine ring adopts a chair conformation. The 1,3,4-oxadiazole-2(3*H*)-thione group makes dihedral angles of 78.69 (8), 53.56 (7) and 55.30 (9)° with the benzene rings. In the crystal, O—H⋯O, C—H⋯S and C—H⋯F hydrogen bonds linked the mol­ecules into layers lying parallel to the *ab* plane. Weak C—H⋯π inter­actions also occur.

## Related literature

For pharmacological background, see: Bhatia & Gupta (2011 ▶); Liu (2006 ▶). For ring conformations, see: Cremer & Pople (1975 ▶). For bond-length data, see: Allen *et al.* (1987 ▶).
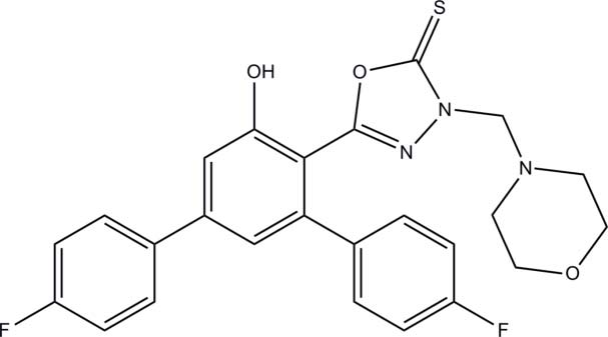


## Experimental

### 

#### Crystal data

C_25_H_21_F_2_N_3_O_3_S
*M* *_r_* = 481.51
Monoclinic, 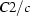

*a* = 16.0547 (14) Å
*b* = 11.4125 (11) Å
*c* = 25.364 (2) Å
β = 94.202 (2)°
*V* = 4634.9 (7) Å^3^
*Z* = 8
Mo *K*α radiation
μ = 0.19 mm^−1^
*T* = 296 K
0.48 × 0.25 × 0.17 mm

#### Data collection

Bruker SMART APEXII DUO CCD area-detector diffractometer
Absorption correction: multi-scan (*SADABS*; Bruker, 2009 ▶) *T*_min_ = 0.916, *T*_max_ = 0.969
23057 measured reflections
6182 independent reflections
4139 reflections with *I* > 2σ(*I*)
*R*_int_ = 0.029

#### Refinement

*R*[*F* ^2^ > 2σ(*F* ^2^)] = 0.044
*wR*(*F* ^2^) = 0.143
*S* = 1.03
6182 reflections
311 parameters
H atoms treated by a mixture of independent and constrained refinement
Δρ_max_ = 0.24 e Å^−3^
Δρ_min_ = −0.24 e Å^−3^

### 

Data collection: *APEX2* (Bruker, 2009 ▶); cell refinement: *SAINT* (Bruker, 2009 ▶); data reduction: *SAINT*; program(s) used to solve structure: *SHELXTL* (Sheldrick, 2008 ▶); program(s) used to refine structure: *SHELXTL*; molecular graphics: *SHELXTL*; software used to prepare material for publication: *SHELXTL* and *PLATON* (Spek, 2009 ▶).

## Supplementary Material

Crystal structure: contains datablock(s) global, I. DOI: 10.1107/S1600536811048471/hb6502sup1.cif

Structure factors: contains datablock(s) I. DOI: 10.1107/S1600536811048471/hb6502Isup2.hkl

Supplementary material file. DOI: 10.1107/S1600536811048471/hb6502Isup3.cml

Additional supplementary materials:  crystallographic information; 3D view; checkCIF report

## Figures and Tables

**Table 1:** Hydrogen-bond geometry (Å, °) *Cg*1 is the centroid of the C7–C12 ring.

*D*—H⋯*A*	*D*—H	H⋯*A*	*D*⋯*A*	*D*—H⋯*A*
O3—H1*O*3⋯O2^i^	0.79 (2)	1.95 (2)	2.728 (2)	167 (2)
C5—H5*A*⋯S1^ii^	0.93	2.80	3.639 (2)	151
C12—H12*A*⋯F1^iii^	0.93	2.47	3.292 (2)	148
C23—H23*A*⋯F1^iv^	0.97	2.51	3.462 (3)	167
C1—H1*A*⋯*Cg*1^v^	0.93	2.91	3.414 (2)	115

Symmetry codes: (i) 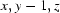; (ii) 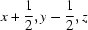; (iii) 
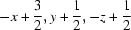
; (iv) 
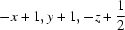
; (v) 
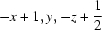
.

**Fig. 1.**
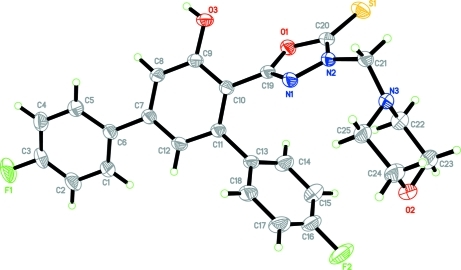
The molecular structure of the title compound with 30% probability displacement ellipsoids.

**Fig. 2.**
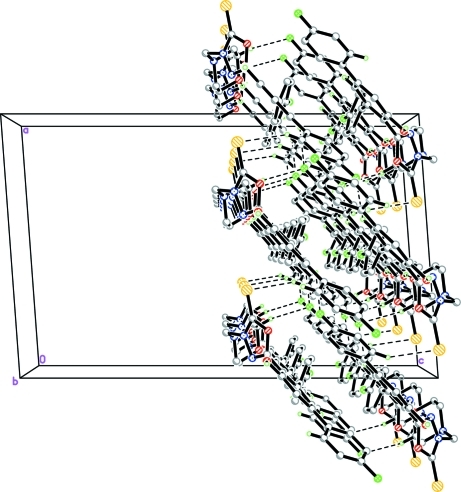
The crystal packing of the title compound. Dashed lines represent the hydrogen bonds. Hydrogen atoms not involved in hydrogen bonding have been omitted for the sake of clarity.

## supplementary crystallographic information

### Comment

Substituted 1,3,4-oxadiazoles are of considerable pharmaceutical interest.  For a recent review, see: Bhatia & Gupta (2011). Polysubstituted aromatics such as terphenyls exhibit considerable biological properties, *e.g.*, potent anticoagulant, immunosuppressants, antithrombotic, neuroprotective, specific 5-lipoxygenase inhibitory and cytotoxic activities (Liu, 2006). Encouraged by these diverse biological activities of 1,3,4-oxadiazoles and terphenyls, our research group has decided to prepare terphenyl derivative bearing the oxadiazole moiety, thus bringing both types of functional groups together in a single molecule: we now report the synthesis and structure of the title compound, (I). The precursor of the title compound was prepared from 4,4'-difluorochalcone by several steps.

The molecular structure is shown in Fig. 1. The morpholine ring adopts a chair conformation with puckering parameters Q= 0.579 (2) Å, Θ= 2.9 (2)° and φ= 247 (5)° (Cremer & Pople, 1975). The 1,3,4-oxadiazole-2(3*H*)-thione (S1/C20/O1/C19/N1/N2) group makes dihedral angles of 78.69 (8), 53.56 (7) and 55.30 (9) ° with the benzene (C1–C6, C7–C12 & C13–C18) rings, respectively. Bond lengths (Allen *et al.*, 1987) and angles are within normal ranges.

The crystal packing is shown in Fig. 2. Intermolecular O3—H1O3···O2, C5—H5A···S1, C12—H12A···F1 and C23—H23A···F1 hydrogen bonds (Table 1) linked the molecules into layers parallel to *ab* plane. C—H···π interactions (Table 1) which involves C1 and phenyl ring (*Cg*1 = C7–C12) further stabilize the crystal structure.

### Experimental

To a solution of 5-(4,4''-difluoro-5'-hydroxy-1,1':3',1''-terphenyl-4'-yl) -1,3,4-oxadiazole-2(3*H*)-thione (3.82 g, 0.01 mol) in ethanol (5 ml) was added formaldehyde (0.5 ml, 37%) and morpholine (0.01 mol). The reaction mixture was stirred overnight. After cooling, the precipitate was filtered and crystallized from ethanol.  Colourless blocks of (I) were grown from 1:1 mixture of ethanol and DMF by slow evaporation and the yield was 72%. *M.p.*: 475 K.

### Refinement

H1O3 atom attached to the O atom was located from the difference map and refined freely, [O–H = 0.78 (3) Å]. The remaining H atoms were positioned geometrically [C–H = 0.93 or 0.97 Å] and refined using a riding model with *U*_iso_(H) = 1.2 *U*_eq_(C).

### Crystal data

**Table:**

C_25_H_21_F_2_N_3_O_3_S	*F*(000) = 2000
*M**_r_* = 481.51	*D*_x_ = 1.380 Mg m^−^^3^
Monoclinic, *C*2/*c*	Mo *K*α radiation, λ = 0.71073 Å
Hall symbol: -C 2yc	Cell parameters from 6002 reflections
*a* = 16.0547 (14) Å	θ = 2.3–27.3°
*b* = 11.4125 (11) Å	µ = 0.19 mm^−^^1^
*c* = 25.364 (2) Å	*T* = 296 K
β = 94.202 (2)°	Block, colourless
*V* = 4634.9 (7) Å^3^	0.48 × 0.25 × 0.17 mm
*Z* = 8	

### Data collection

**Table:**

Bruker SMART APEXII DUO CCD area-detector diffractometer	6182 independent reflections
Radiation source: fine-focus sealed tube	4139 reflections with *I* > 2σ(*I*)
graphite	*R*_int_ = 0.029
φ and ω scans	θ_max_ = 29.1°, θ_min_ = 1.6°
Absorption correction: multi-scan (*SADABS*; Bruker, 2009)	*h* = −20→21
*T*_min_ = 0.916, *T*_max_ = 0.969	*k* = −15→14
23057 measured reflections	*l* = −34→34

### Refinement

**Table:**

Refinement on *F*^2^	Primary atom site location: structure-invariant direct methods
Least-squares matrix: full	Secondary atom site location: difference Fourier map
*R*[*F*^2^ > 2σ(*F*^2^)] = 0.044	Hydrogen site location: inferred from neighbouring sites
*wR*(*F*^2^) = 0.143	H atoms treated by a mixture of independent and constrained refinement
*S* = 1.03	*w* = 1/[σ^2^(*F*_o_^2^) + (0.0688*P*)^2^ + 1.531*P*] where *P* = (*F*_o_^2^ + 2*F*_c_^2^)/3
6182 reflections	(Δ/σ)_max_ < 0.001
311 parameters	Δρ_max_ = 0.24 e Å^−^^3^
0 restraints	Δρ_min_ = −0.24 e Å^−^^3^

### Special details

**Table:**

Geometry. All e.s.d.'s (except the e.s.d. in the dihedral angle between two l.s. planes) are estimated using the full covariance matrix. The cell e.s.d.'s are taken into account individually in the estimation of e.s.d.'s in distances, angles and torsion angles; correlations between e.s.d.'s in cell parameters are only used when they are defined by crystal symmetry. An approximate (isotropic) treatment of cell e.s.d.'s is used for estimating e.s.d.'s involving l.s. planes.
Refinement. Refinement of *F*^2^ against ALL reflections. The weighted *R*-factor *wR* and goodness of fit *S* are based on *F*^2^, conventional *R*-factors *R* are based on *F*, with *F* set to zero for negative *F*^2^. The threshold expression of *F*^2^ > σ(*F*^2^) is used only for calculating *R*-factors(gt) *etc*. and is not relevant to the choice of reflections for refinement. *R*-factors based on *F*^2^ are statistically about twice as large as those based on *F*, and *R*- factors based on ALL data will be even larger.

### Fractional atomic coordinates and isotropic or equivalent isotropic displacement parameters (Å^2^)

**Table:**

	*x*	*y*	*z*	*U*_iso_*/*U*_eq_	
S1	0.15583 (3)	0.82030 (5)	0.44595 (2)	0.06259 (17)	
F1	0.77333 (9)	0.33903 (13)	0.20221 (6)	0.0943 (5)	
F2	0.46943 (11)	1.25156 (10)	0.31200 (6)	0.1043 (5)	
O1	0.29532 (7)	0.74319 (10)	0.40602 (4)	0.0469 (3)	
O2	0.38526 (11)	1.29292 (12)	0.42547 (7)	0.0812 (5)	
O3	0.39200 (10)	0.53070 (12)	0.41580 (6)	0.0697 (5)	
N1	0.39553 (9)	0.85596 (11)	0.44436 (5)	0.0439 (3)	
N2	0.31768 (8)	0.88887 (11)	0.45963 (5)	0.0420 (3)	
N3	0.32987 (9)	1.09806 (11)	0.48147 (6)	0.0462 (3)	
C1	0.64272 (12)	0.56922 (18)	0.24724 (7)	0.0583 (5)	
H1A	0.6243	0.6433	0.2366	0.070*	
C2	0.69619 (13)	0.5084 (2)	0.21659 (8)	0.0673 (6)	
H2A	0.7141	0.5411	0.1858	0.081*	
C3	0.72171 (12)	0.4003 (2)	0.23261 (8)	0.0616 (5)	
C4	0.69747 (13)	0.34894 (18)	0.27745 (9)	0.0635 (5)	
H4A	0.7161	0.2744	0.2873	0.076*	
C5	0.64462 (12)	0.41028 (16)	0.30795 (8)	0.0569 (5)	
H5A	0.6277	0.3764	0.3388	0.068*	
C6	0.61605 (10)	0.52180 (14)	0.29362 (6)	0.0456 (4)	
C7	0.55723 (10)	0.58586 (14)	0.32581 (6)	0.0451 (4)	
C8	0.50339 (11)	0.52572 (14)	0.35719 (7)	0.0504 (4)	
H8A	0.5062	0.4445	0.3594	0.061*	
C9	0.44575 (11)	0.58574 (14)	0.38513 (7)	0.0498 (4)	
C10	0.43949 (10)	0.70772 (13)	0.38186 (6)	0.0432 (4)	
C11	0.49340 (10)	0.76946 (13)	0.35017 (6)	0.0428 (4)	
C12	0.55155 (11)	0.70777 (14)	0.32334 (7)	0.0473 (4)	
H12A	0.5878	0.7488	0.3031	0.057*	
C13	0.48645 (11)	0.89862 (13)	0.34198 (6)	0.0442 (4)	
C14	0.41390 (13)	0.94776 (16)	0.31948 (8)	0.0605 (5)	
H14A	0.3680	0.8998	0.3111	0.073*	
C15	0.40759 (16)	1.06664 (18)	0.30901 (9)	0.0715 (6)	
H15A	0.3585	1.0987	0.2935	0.086*	
C16	0.47502 (17)	1.13499 (16)	0.32200 (9)	0.0669 (6)	
C17	0.54758 (16)	1.09168 (18)	0.34457 (10)	0.0725 (6)	
H17A	0.5925	1.1412	0.3533	0.087*	
C18	0.55406 (13)	0.97158 (16)	0.35447 (8)	0.0591 (5)	
H18A	0.6038	0.9405	0.3695	0.071*	
C19	0.37959 (10)	0.77082 (13)	0.41218 (6)	0.0410 (3)	
C20	0.25662 (11)	0.82095 (14)	0.43819 (6)	0.0434 (4)	
C21	0.30977 (12)	0.98278 (14)	0.49890 (7)	0.0497 (4)	
H21A	0.2528	0.9833	0.5093	0.060*	
H21B	0.3460	0.9643	0.5301	0.060*	
C22	0.28404 (13)	1.13579 (17)	0.43316 (8)	0.0602 (5)	
H22A	0.2249	1.1204	0.4352	0.072*	
H22B	0.3030	1.0927	0.4033	0.072*	
C23	0.29823 (15)	1.26587 (18)	0.42564 (9)	0.0719 (6)	
H23A	0.2698	1.2908	0.3925	0.086*	
H23B	0.2744	1.3088	0.4539	0.086*	
C24	0.42904 (15)	1.25413 (18)	0.47370 (12)	0.0814 (7)	
H24A	0.4074	1.2946	0.5034	0.098*	
H24B	0.4879	1.2729	0.4731	0.098*	
C25	0.41867 (12)	1.12362 (16)	0.48025 (10)	0.0644 (5)	
H25A	0.4414	1.0825	0.4510	0.077*	
H25B	0.4484	1.0978	0.5129	0.077*	
H1O3	0.3975 (15)	0.462 (2)	0.4167 (10)	0.085 (8)*	

### Atomic displacement parameters (Å^2^)

**Table:**

	*U*^11^	*U*^22^	*U*^33^	*U*^12^	*U*^13^	*U*^23^
S1	0.0450 (3)	0.0795 (4)	0.0650 (3)	−0.0045 (2)	0.0151 (2)	0.0078 (2)
F1	0.0814 (9)	0.1175 (12)	0.0876 (9)	0.0413 (8)	0.0312 (7)	−0.0178 (8)
F2	0.1615 (16)	0.0372 (6)	0.1162 (11)	−0.0069 (7)	0.0247 (11)	0.0151 (7)
O1	0.0495 (7)	0.0439 (6)	0.0489 (6)	−0.0027 (5)	0.0138 (5)	−0.0051 (5)
O2	0.0963 (12)	0.0428 (7)	0.1116 (12)	0.0026 (7)	0.0566 (10)	0.0151 (8)
O3	0.0916 (11)	0.0357 (7)	0.0894 (10)	−0.0003 (7)	0.0576 (9)	0.0079 (6)
N1	0.0444 (7)	0.0367 (7)	0.0522 (7)	−0.0046 (5)	0.0149 (6)	−0.0034 (6)
N2	0.0448 (8)	0.0333 (6)	0.0500 (7)	−0.0026 (5)	0.0170 (6)	−0.0023 (5)
N3	0.0476 (8)	0.0344 (7)	0.0585 (8)	0.0001 (6)	0.0167 (6)	−0.0009 (6)
C1	0.0570 (11)	0.0596 (11)	0.0609 (10)	0.0109 (9)	0.0209 (9)	0.0060 (9)
C2	0.0602 (13)	0.0873 (15)	0.0574 (10)	0.0148 (11)	0.0241 (9)	0.0043 (10)
C3	0.0450 (10)	0.0789 (14)	0.0621 (11)	0.0153 (9)	0.0114 (8)	−0.0157 (10)
C4	0.0587 (12)	0.0581 (11)	0.0748 (13)	0.0153 (9)	0.0117 (10)	−0.0077 (10)
C5	0.0599 (11)	0.0527 (10)	0.0597 (10)	0.0059 (8)	0.0163 (9)	0.0002 (8)
C6	0.0416 (9)	0.0453 (9)	0.0509 (8)	0.0004 (7)	0.0105 (7)	−0.0044 (7)
C7	0.0450 (9)	0.0416 (8)	0.0501 (8)	−0.0016 (7)	0.0129 (7)	−0.0034 (7)
C8	0.0615 (11)	0.0322 (8)	0.0603 (10)	0.0012 (7)	0.0230 (8)	0.0000 (7)
C9	0.0603 (11)	0.0364 (8)	0.0557 (9)	−0.0037 (7)	0.0249 (8)	0.0004 (7)
C10	0.0481 (9)	0.0341 (8)	0.0492 (8)	−0.0036 (6)	0.0165 (7)	−0.0048 (6)
C11	0.0440 (9)	0.0343 (8)	0.0513 (8)	−0.0074 (6)	0.0123 (7)	−0.0036 (6)
C12	0.0454 (9)	0.0416 (8)	0.0569 (9)	−0.0088 (7)	0.0179 (8)	−0.0020 (7)
C13	0.0510 (10)	0.0348 (8)	0.0488 (8)	−0.0092 (7)	0.0174 (7)	−0.0038 (6)
C14	0.0619 (12)	0.0458 (10)	0.0732 (12)	−0.0109 (9)	0.0013 (10)	0.0040 (9)
C15	0.0857 (16)	0.0513 (11)	0.0769 (13)	0.0032 (11)	0.0012 (12)	0.0133 (10)
C16	0.0993 (17)	0.0350 (9)	0.0689 (12)	−0.0083 (10)	0.0234 (12)	0.0032 (8)
C17	0.0840 (16)	0.0447 (11)	0.0906 (15)	−0.0277 (11)	0.0186 (13)	−0.0078 (10)
C18	0.0573 (11)	0.0458 (10)	0.0753 (12)	−0.0151 (8)	0.0122 (9)	−0.0045 (9)
C19	0.0456 (9)	0.0325 (7)	0.0464 (8)	−0.0056 (6)	0.0137 (7)	0.0005 (6)
C20	0.0472 (9)	0.0408 (8)	0.0434 (8)	−0.0030 (7)	0.0127 (7)	0.0057 (6)
C21	0.0621 (11)	0.0389 (8)	0.0509 (9)	−0.0035 (7)	0.0232 (8)	−0.0050 (7)
C22	0.0640 (12)	0.0498 (10)	0.0677 (12)	0.0024 (9)	0.0110 (10)	0.0033 (9)
C23	0.0871 (16)	0.0531 (11)	0.0778 (13)	0.0152 (11)	0.0208 (12)	0.0155 (10)
C24	0.0629 (13)	0.0455 (11)	0.139 (2)	−0.0091 (9)	0.0271 (15)	0.0061 (13)
C25	0.0505 (11)	0.0415 (10)	0.1024 (16)	−0.0016 (8)	0.0153 (10)	0.0027 (10)

### Geometric parameters (Å, °)

**Table:**

S1—C20	1.6445 (17)	C8—H8A	0.9300
F1—C3	1.365 (2)	C9—C10	1.398 (2)
F2—C16	1.356 (2)	C10—C11	1.412 (2)
O1—C20	1.3834 (18)	C10—C19	1.464 (2)
O1—C19	1.3868 (19)	C11—C12	1.387 (2)
O2—C23	1.431 (3)	C11—C13	1.492 (2)
O2—C24	1.436 (3)	C12—H12A	0.9300
O3—C9	1.3577 (18)	C13—C14	1.378 (3)
O3—H1O3	0.78 (3)	C13—C18	1.386 (2)
N1—C19	1.283 (2)	C14—C15	1.385 (3)
N1—N2	1.3871 (18)	C14—H14A	0.9300
N2—C20	1.334 (2)	C15—C16	1.355 (3)
N2—C21	1.4749 (19)	C15—H15A	0.9300
N3—C21	1.432 (2)	C16—C17	1.353 (3)
N3—C22	1.448 (3)	C17—C18	1.396 (3)
N3—C25	1.458 (2)	C17—H17A	0.9300
C1—C2	1.386 (2)	C18—H18A	0.9300
C1—C6	1.391 (2)	C21—H21A	0.9700
C1—H1A	0.9300	C21—H21B	0.9700
C2—C3	1.353 (3)	C22—C23	1.516 (3)
C2—H2A	0.9300	C22—H22A	0.9700
C3—C4	1.362 (3)	C22—H22B	0.9700
C4—C5	1.380 (2)	C23—H23A	0.9700
C4—H4A	0.9300	C23—H23B	0.9700
C5—C6	1.392 (2)	C24—C25	1.509 (3)
C5—H5A	0.9300	C24—H24A	0.9700
C6—C7	1.485 (2)	C24—H24B	0.9700
C7—C12	1.395 (2)	C25—H25A	0.9700
C7—C8	1.397 (2)	C25—H25B	0.9700
C8—C9	1.387 (2)		
			
C20—O1—C19	105.32 (12)	C13—C14—H14A	119.1
C23—O2—C24	110.36 (15)	C15—C14—H14A	119.1
C9—O3—H1O3	113.7 (18)	C16—C15—C14	118.1 (2)
C19—N1—N2	103.95 (13)	C16—C15—H15A	120.9
C20—N2—N1	112.27 (12)	C14—C15—H15A	120.9
C20—N2—C21	126.91 (14)	C17—C16—C15	122.69 (18)
N1—N2—C21	120.62 (13)	C17—C16—F2	118.7 (2)
C21—N3—C22	114.98 (15)	C15—C16—F2	118.6 (2)
C21—N3—C25	115.67 (14)	C16—C17—C18	119.03 (19)
C22—N3—C25	111.09 (15)	C16—C17—H17A	120.5
C2—C1—C6	121.44 (18)	C18—C17—H17A	120.5
C2—C1—H1A	119.3	C13—C18—C17	120.1 (2)
C6—C1—H1A	119.3	C13—C18—H18A	119.9
C3—C2—C1	118.32 (18)	C17—C18—H18A	119.9
C3—C2—H2A	120.8	N1—C19—O1	113.05 (13)
C1—C2—H2A	120.8	N1—C19—C10	126.79 (15)
C2—C3—C4	123.01 (17)	O1—C19—C10	120.12 (13)
C2—C3—F1	118.72 (18)	N2—C20—O1	105.36 (13)
C4—C3—F1	118.27 (19)	N2—C20—S1	130.84 (12)
C3—C4—C5	118.36 (19)	O1—C20—S1	123.79 (12)
C3—C4—H4A	120.8	N3—C21—N2	115.25 (13)
C5—C4—H4A	120.8	N3—C21—H21A	108.5
C4—C5—C6	121.45 (17)	N2—C21—H21A	108.5
C4—C5—H5A	119.3	N3—C21—H21B	108.5
C6—C5—H5A	119.3	N2—C21—H21B	108.5
C1—C6—C5	117.43 (15)	H21A—C21—H21B	107.5
C1—C6—C7	121.40 (15)	N3—C22—C23	109.02 (18)
C5—C6—C7	121.14 (15)	N3—C22—H22A	109.9
C12—C7—C8	118.31 (14)	C23—C22—H22A	109.9
C12—C7—C6	120.54 (14)	N3—C22—H22B	109.9
C8—C7—C6	121.07 (14)	C23—C22—H22B	109.9
C9—C8—C7	120.80 (15)	H22A—C22—H22B	108.3
C9—C8—H8A	119.6	O2—C23—C22	111.55 (16)
C7—C8—H8A	119.6	O2—C23—H23A	109.3
O3—C9—C8	122.70 (15)	C22—C23—H23A	109.3
O3—C9—C10	116.67 (14)	O2—C23—H23B	109.3
C8—C9—C10	120.62 (14)	C22—C23—H23B	109.3
C9—C10—C11	119.10 (14)	H23A—C23—H23B	108.0
C9—C10—C19	120.39 (13)	O2—C24—C25	110.3 (2)
C11—C10—C19	120.48 (14)	O2—C24—H24A	109.6
C12—C11—C10	119.26 (14)	C25—C24—H24A	109.6
C12—C11—C13	118.65 (13)	O2—C24—H24B	109.6
C10—C11—C13	121.99 (13)	C25—C24—H24B	109.6
C11—C12—C7	121.89 (14)	H24A—C24—H24B	108.1
C11—C12—H12A	119.1	N3—C25—C24	108.40 (16)
C7—C12—H12A	119.1	N3—C25—H25A	110.0
C14—C13—C18	118.30 (16)	C24—C25—H25A	110.0
C14—C13—C11	120.80 (15)	N3—C25—H25B	110.0
C18—C13—C11	120.83 (17)	C24—C25—H25B	110.0
C13—C14—C15	121.73 (19)	H25A—C25—H25B	108.4
			
C19—N1—N2—C20	2.31 (17)	C18—C13—C14—C15	0.4 (3)
C19—N1—N2—C21	177.47 (14)	C11—C13—C14—C15	−176.60 (18)
C6—C1—C2—C3	0.5 (3)	C13—C14—C15—C16	−0.6 (3)
C1—C2—C3—C4	−0.2 (3)	C14—C15—C16—C17	0.1 (3)
C1—C2—C3—F1	178.97 (19)	C14—C15—C16—F2	−179.89 (19)
C2—C3—C4—C5	−0.2 (3)	C15—C16—C17—C18	0.7 (3)
F1—C3—C4—C5	−179.36 (19)	F2—C16—C17—C18	−179.34 (19)
C3—C4—C5—C6	0.2 (3)	C14—C13—C18—C17	0.4 (3)
C2—C1—C6—C5	−0.5 (3)	C11—C13—C18—C17	177.39 (17)
C2—C1—C6—C7	−178.59 (18)	C16—C17—C18—C13	−0.9 (3)
C4—C5—C6—C1	0.1 (3)	N2—N1—C19—O1	−1.38 (17)
C4—C5—C6—C7	178.19 (18)	N2—N1—C19—C10	176.19 (14)
C1—C6—C7—C12	−24.7 (3)	C20—O1—C19—N1	0.08 (17)
C5—C6—C7—C12	157.23 (18)	C20—O1—C19—C10	−177.68 (13)
C1—C6—C7—C8	152.03 (18)	C9—C10—C19—N1	125.76 (19)
C5—C6—C7—C8	−26.0 (3)	C11—C10—C19—N1	−52.3 (2)
C12—C7—C8—C9	0.0 (3)	C9—C10—C19—O1	−56.8 (2)
C6—C7—C8—C9	−176.80 (17)	C11—C10—C19—O1	125.07 (16)
C7—C8—C9—O3	179.61 (18)	N1—N2—C20—O1	−2.28 (16)
C7—C8—C9—C10	0.9 (3)	C21—N2—C20—O1	−177.07 (14)
O3—C9—C10—C11	−179.50 (17)	N1—N2—C20—S1	176.89 (12)
C8—C9—C10—C11	−0.8 (3)	C21—N2—C20—S1	2.1 (2)
O3—C9—C10—C19	2.4 (3)	C19—O1—C20—N2	1.33 (15)
C8—C9—C10—C19	−178.88 (17)	C19—O1—C20—S1	−177.92 (12)
C9—C10—C11—C12	−0.4 (3)	C22—N3—C21—N2	54.9 (2)
C19—C10—C11—C12	177.73 (16)	C25—N3—C21—N2	−76.8 (2)
C9—C10—C11—C13	175.85 (17)	C20—N2—C21—N3	−116.90 (18)
C19—C10—C11—C13	−6.0 (3)	N1—N2—C21—N3	68.7 (2)
C10—C11—C12—C7	1.4 (3)	C21—N3—C22—C23	168.57 (15)
C13—C11—C12—C7	−174.96 (17)	C25—N3—C22—C23	−57.65 (19)
C8—C7—C12—C11	−1.2 (3)	C24—O2—C23—C22	−57.5 (2)
C6—C7—C12—C11	175.63 (16)	N3—C22—C23—O2	56.2 (2)
C12—C11—C13—C14	116.54 (19)	C23—O2—C24—C25	59.3 (2)
C10—C11—C13—C14	−59.7 (2)	C21—N3—C25—C24	−166.88 (18)
C12—C11—C13—C18	−60.4 (2)	C22—N3—C25—C24	59.7 (2)
C10—C11—C13—C18	123.36 (18)	O2—C24—C25—N3	−59.7 (2)

### Hydrogen-bond geometry (Å, °)

**Table:**

Cg1 is the centroid of the C7–C12 ring.

**Table:**

*D*—H···*A*	*D*—H	H···*A*	*D*···*A*	*D*—H···*A*
O3—H1O3···O2^i^	0.79 (2)	1.95 (2)	2.728 (2)	167 (2)
C5—H5A···S1^ii^	0.93	2.80	3.639 (2)	151
C12—H12A···F1^iii^	0.93	2.47	3.292 (2)	148
C23—H23A···F1^iv^	0.97	2.51	3.462 (3)	167
C1—H1A···Cg1^v^	0.93	2.91	3.414 (2)	115

Symmetry codes: (i) *x*, *y*−1, *z*; (ii) *x*+1/2, *y*−1/2, *z*; (iii) −*x*+3/2, *y*+1/2, −*z*+1/2; (iv) −*x*+1, *y*+1, −*z*+1/2; (v) −*x*+1, *y*, −*z*+1/2.
